# National Gay Men’s HIV/AIDS Awareness Day — September 27, 2014

**Published:** 2014-09-26

National Gay Men’s HIV/AIDS Awareness Day is observed each year on September 27 to direct attention to the continuing and disproportionate impact of human immunodeficiency virus infection (HIV) and acquired immune deficiency syndrome (AIDS) on gay, bisexual, and other men who have sex with men (MSM) in the United States. MSM represent approximately 2% of the U.S. population (1); however, in 2010, 63% of all new HIV infections were among MSM (2).

By the end of 2010, an estimated 596,600 MSM were living with HIV infection, 52% of the persons living with HIV infection in the United States (3). In 2011, a report noted that the percentage of MSM who were HIV-positive but unaware of their status was high, even among those recently tested (4).

CDC supports a range of efforts to reduce HIV infection among MSM, including prevention services that increase diagnosis of HIV infection, support the linkage and engagement of MSM in care and treatment, and reduce the risk for acquiring and transmitting HIV. Additional information about these efforts is available at http://www.cdc.gov/hiv/risk/gender/msm. Additional information about National Gay Men’s HIV/AIDS Awareness Day is available at http://www.cdc.gov/features/ngmhaad.
